# Neutrophil-to-lymphocyte Ratio (NLR) as a predictor for recurrence in patients with stage III melanoma

**DOI:** 10.1038/s41598-018-22425-3

**Published:** 2018-03-06

**Authors:** Junjie Ma, James Kuzman, Abhijit Ray, Benjamin O. Lawson, Brian Khong, Si Xuan, Andrew W. Hahn, Hung T. Khong

**Affiliations:** 1 Department of Pharmacotherapy, University of Utah College of Pharmacy, Salt Lake City, UT USA; 2 Sanford USD Medical Center, Sioux Falls, SD USA; 3 Division of Oncology, Huntsman Cancer Institute, University of Utah, Salt Lake City, UT USA; 4 HonorHealth Scottsdale Shea Medical Center, Scottsdale, AZ USA; 5 Adventist Health White Memorial, Los Angeles, CA USA; 6 Leonard D. Schaeffer Center for Health Policy and Economics, University of Southern California, Los Angeles, CA USA; 7 Department of Internal Medicine, University of Utah School of Medicine, Salt Lake City, UT USA; 8 Department of Breast Oncology, Moffitt Cancer Center, Tampa, FL USA

## Abstract

Neutrophil-to-lymphocyte ratio is a strong predictor for overall survival and disease free survival in many cancers. Our study is the first investigation aiming to determine the predictive value of neutrophil-to-lymphocyte ratio on prognosis of patients with stage III melanoma. This retrospective study utilized a cohort of 107 patients with stage III melanoma treated at Huntsman Cancer Institute, University of Utah, from May 2002 to March 2016. The optimal cutoff of neutrophil-to-lymphocyte ratio was determined by the significance of log-rank tests. A total of 97 log-rank tests were conducted to find the optimal cutoff. Disease free survival was assessed using the Kaplan–Meier method, and univariable and multivariable Cox models were applied to evaluate the predictive value of neutrophil-to-lymphocyte ratio. 2.5 was identified as the optimal cutoff. Kaplan–Meier curve showed that the disease free survival rate of the low value group was significantly higher compared to that of high value group. After adjusting for confounders and other prognostic factors, the neutrophil-to-lymphocyte ratio ≥ 2.5 remained a strong predictor for disease recurrence in patients with stage III melanoma.

## Introduction

Melanoma kills an estimated 10,130 people in the United States annually^1^. There are five stages of melanoma (Stage 0, Stage I, Stage II, Stage III, and Stage IV). Stage III melanoma patients include those with regional metastases, either in the regional lymph nodes or intralymphatic metastases manifesting as either satellite or in-transit metastases^2^. There are three definable subgroups with statistically significant differences in survival. Patients with Stage IIIA have 5- survival rates of 78%. Patients with Stage IIIB (any thickness, with one to three lymph nodes involved and ulceration) have a 5-year survival rate of 59%. Patients with Stage IIIC (defined as one to three macroscopic lymph node metastases and ulcerated primary melanoma) were found to have a 5- year survival rate at 40%^2^.

Romano *et al*. researched the site and timing of the first relapse in Stage III melanoma and found that for stage IIIA and IIIB patients, lung and liver were the most common first sites of systemic relapse whereas in stage IIIC patients, first sites of systemic relapses were evenly distributed among skin/subcutaneous, nodal, lung, liver, brain and bone. Within their study, central nervous system recurrence of melanoma was found in 4% of stage IIIA and 7% and 13% in stage IIIB and stage IIIC, respectively. Most importantly, Romano *et al*. found the estimate 5-year survivals for stages IIIA, IIIB, and IIIC from time of first relapse were 20%, 20%, and 11%, respectively^3^. Therefore, it is of the utmost importance to utilize another tool to predict recurrence in stage III melanoma patients.

Neutrophil-to-lymphocyte ratio (NLR) integrated information on the inflammatory milieu and physiological stress, which makes it a strong predictor for prognosis in patients with different diseases. Prior studies have shown that increased NLR is associated with decreased overall survival (OS) and disease free survival (DFS) in melanoma, gastric cancer, breast cancer, lung cancer, and gastrointestinal cancer^4–9^. For example, Lino-Silva *et al*. reported that basal NLR ≥ 2 is associated with decreased OS in stage II melanoma patients^4^. Shimada *et al*. concluded a high preoperative NLR may be a convenient biomarker to identify patients with a poor prognosis after resection for primary gastric cancer^5^. Azab *et al*. discovered NLR is an independent predictor of short- and long-term mortality in breast cancer patients with NLR > 3.3^6^. Tomita *et al*. found a high preoperative NLR may be a convenient biomarker to identify patients with a poor prognosis after resection for non-small cell lung cancer^7^. Halazun *et al*. noticed an elevated NLR increases both risk of death and the risk of recurrence in patients who undergo surgery for colorectal liver metastases^8^. Moreover, a meta-analysis based 144 studies suggested that NLR is a significant prognostic indicator in gastrointestinal cancers^9^.

Most of previous studies in melanoma focused on patients with advanced metastatic disease^10,11^. A retrospective cohort study conducted by Ferrucci *et al*. showed that baseline NLR was significantly associated with survival in patients treated with ipilimumab^10^. Similarly, Zaragoza *et al*. also conducted a retrospective cohort study in melanoma patients treated with ipilimumab^11^. They reported NLR is an independent prognostic indicator of poor survival in metastatic melanoma patients. The treatment and prognosis of stage III patients are very different from stage IV patients whose melanoma has spread to distant areas of body.

A few studies estimated the association between NLR and long-term survival^4,12^. Lino-Silva reported that elevated NLR was associated with decreased 5-year OS in stage II melanoma patients^4^. Davis *et al*. found that NLR was independently associated with disease-specific death in patients with nonmetastatic melanoma^12^. However, it is still unclear whether high NLR is a passive indicator of recurrence in stage III melanoma patients.

This is the first study that used NLR to predict the DFS in stage III melanoma patients. The aim of our study was to determine the predictive value of NLR on disease prognosis in stage III melanoma patients.

## Results

**Figure 1:**
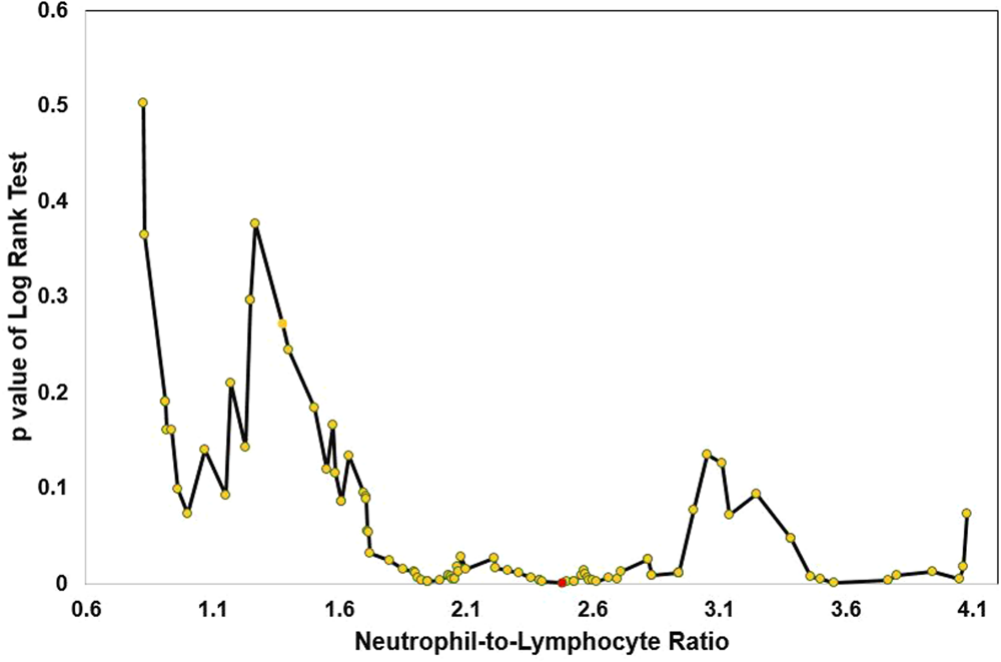
Association between NLR value and p-value of log-rank tests.

A total of 107 patients were enrolled in this study. The baseline characteristics are presented in Table 1. We conducted 97 log-rank tests to determine the optimal cutoff.The association between NLR value and p-value of log-rank tests are presented in Fig. 1. The NLR cutoff value was determined at 2.5.

**Table 1:** Baseline characteristics of stage III melanoma patients according to the NLR.

	No. (%)		No. (%)		No. (%)		p-value
	Total (N = 107)		NLR < 2.5 (N = 60)		NLR ≥ 2.5 (N = 47)		
**Age of diagnosis (years)**							0.435
<35	23	(21.5)	14	(23.3)	9	(19.2)	
35–49	22	(20.6)	15	(25.0)	7	(14.9)	
50–64	38	(35.5)	20	(33.3)	18	(38.3)	
65+	24	(22.4)	11	(18.3)	13	(27.7)	
**Gender**							0.066
Female	40	(37.4)	27	(45.0)	13	(27.7)	
Male	67	(62.6)	33	(55.0)	34	(72.3)	
**Site**							0.539
Head and Neck	28	(26.4)	17	(28.3)	11	(23.9)	
Trunk	42	(39.6)	21	(35.0)	21	(45.7)	
Limb	36	(34.0)	22	(36.7)	14	(30.4)	
**BRAF**							0.102
Negative	16	(14.9)	7	(11.7)	9	(19.2)	
Positive	18	(16.8)	7	(11.7)	11	(23.4)	
Unknown	73	(68.2)	46	(76.7)	27	(57.4)	
**Stage III**							0.221
A	33	(31.4)	20	(34.5)	13	(27.7)	
B	44	(41.9)	20	(34.5)	24	(51.1)	
C	28	(26.7)	18	(31.0)	10	(21.3)	
**N stage**							0.748
0	3	(3.0)	2	(3.6)	1	(2.2)	
1	62	(61.4)	36	(65.5)	26	(56.5)	
2	25	(24.8)	12	(21.8)	13	(28.3)	
3	11	(10.9)	5	(9.1)	6	(13.0)	
**Adjuvant therapy**							0.044
No	52	(48.6)	24	(40.0)	28	(59.6)	
Yes	55	(51.4)	36	(60.0)	19	(40.4)	
**Radiation**							0.836
No	61	(57.0)	33	(55.0)	28	(59.6)	
Yes	9	(8.4)	6	(10.0)	3	(6.38)	
Unknown	37	(34.6)	21	(35.0)	16	(34.0)	
**T stage**							0.687
T1	19	(17.8)	13	(21.7)	6	(12.8)	
T2	33	(30.8)	18	(30.0)	15	(31.9)	
T3	22	(20.6)	10	(16.7)	12	(25.5)	
T4	17	(15.9)	10	(16.7)	7	(14.9)	
Unknown	16	(15.0)	9	(15.0)	7	(14.9)	
**Ulceration**							0.280
No	48	(44.9)	24	(40.0)	24	(51.1)	
Yes	44	(41.1)	25	(41.7)	19	(40.4)	
Unknown	15	(14.0)	11	(18.3)	4	(8.5)	
**TIL**							0.177
No	58	(55.2)	36	(61.0)	22	(47.8)	
Yes	47	(44.8)	23	(39.0)	24	(52.2)	
	**Median**	**IQR**	**Median**	**IQR**	**Median**	**IQR**	
**WBC**	6.66	(5.34, 8.33)	6.27	(5.10, 7.62)	7.03	(5.48, 9.01)	0.036
**ANC**	4.30	(3.10, 5.40)	3.55	(2.80, 4.65)	5.00	(3.90, 6.40)	<0.001
**ALC**	1.90	(1.40, 2.30)	2.30	(1.75, 2.55)	1.50	(1.20, 1.90)	<0.001
**NLR**	2.27	(1.71, 3.00)	1.72	(1.27, 2.05)	3.11	(2.70, 4.06)	<0.001
**Breslow**	1.65	(1.15, 3.35)	1.60	(0.93, 3.65)	1.90	(1.25, 3.28)	0.508

*TIL, tumor-infiltrating lymphocytes; WBC, white blood cell counts; ANC, absolute neutrophil count; ALC absolute lymphocyte count; NLR, neutrophil-to-lymphocyte ratio; Breslow, Breslow’s thickness measures in millimeters.

The high NLR group had 47 patients whose NLR was ≥ 2.5, and the low NLR group had 60 patients whose NLR was < 2.5. There were no statistically significant differences in age, gender, site of melanoma, BRAF mutation status, stage IIIA, B or C status, lymph node status (N stage), the tumor size (T stage), ulceration status, tumor-infiltrating lymphocytes (TILs), and the Breslow depth (Table 1).

There were more female patients in the low NLR group compared with the high NLR group. The low NLR group had 27 (45.0%) female patients, while the high NLR group had 13 (27.7%) female patients, although the difference was not significant (p = 0.066). Compared with the high NLR group, the low NLR group had more patients who received adjuvant therapy (36, 60.0% vs. 19, 40.4%, p = 0.044). The white blood cell (WBC) count of the low NLR group was slightly lower than that of the high NLR group [Median 6.27 (±IQR 5.10, 7.62) vs Median 7.03 (±IQR 5.48, 9.01), p = 0.036].

**Figure 2:**
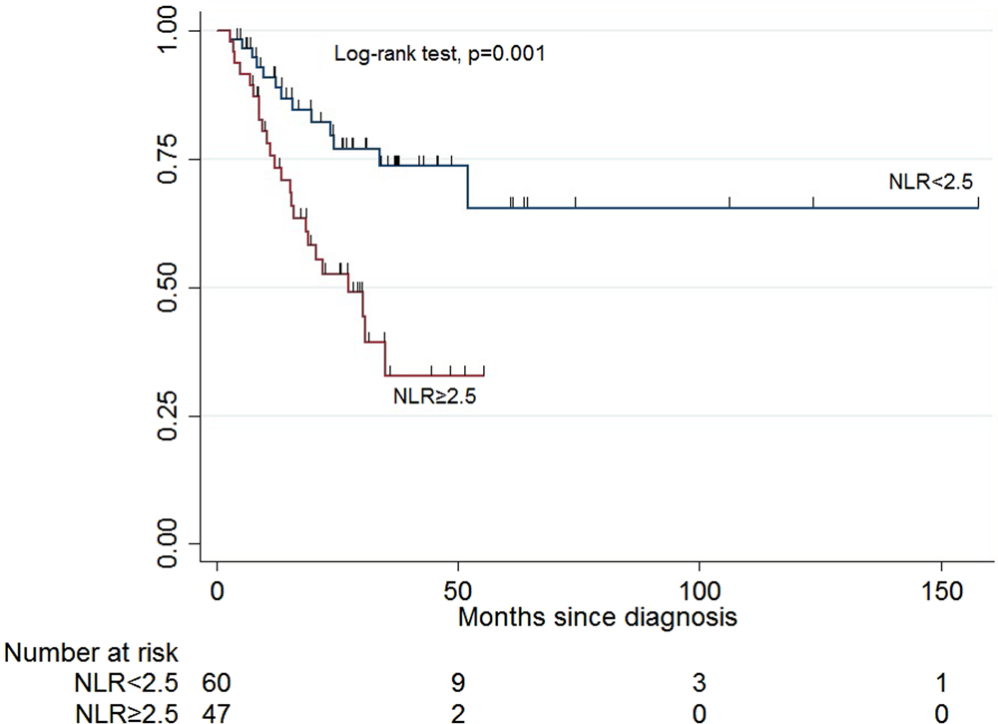
Kaplan-Meier survival curves showing the DFS by NLR.

The median (range) of follow-up period was 21.1 (2.9, 123.2) months. The Kaplan-Meier (KM) curve showed that the DFS rate of the low NLR group was significantly higher compared to that of the high NLR group (Fig. 2). The p-value of log-rank test was 0.001. The univariable model showed that patients with baseline NLR < 2.5 had a significantly improved DFS [Hazard Ratio (HR) 2.99, 95% Confidence Interval (CI) 1.50 to 5.94, p-value = 0.002]. In the multivariable Cox proportional hazard model, we adjusted for age of diagnosis, gender, site of melanoma, stage IIIA, B or C status, lymph node status (N stage), adjuvant therapy, the tumor size (T stage), ulceration status, white blood cell counts, tumor-infiltrating lymphocytes (TILs), and the Breslow depth. The adjusted hazard ratio of DFS for patients with baseline NLR ≥ 2.5 was 3.82 (95% CI 1.26 to 11.56, p-value = 0.018) compared to patients with baseline NLR < 2.5. Schoenfeld’s global test indicated the proportional hazards assumption was not violated (p = 0.409 for the multivariable Cox model). Hazard ratios for the disease free survival are presented in Table 2.

**Table 2:** Hazard ratios for the disease free survival in stage III melanoma patients.

Model	HR	95% CI Lower	95% CI Upper	P-value
Univariable model	2.99	1.50	5.94	0.002
Multivariable model*	3.82	1.26	11.56	0.018

*Adjusted for age of diagnosis, gender, site of melanoma, stage IIIA, B or C status, lymph node status (N stage), adjuvant therapy, the tumor size (T stage), ulceration status, white blood cell counts, tumor-infiltrating lymphocytes (TILs), and the Breslow depth.

## Discussion

To our best knowledge, this is the first study to evaluate the predictive value of NLR on melanoma recurrence in patients diagnosed with stage III melanoma. Our analyses indicated that NLR is a significant predictor for recurrence in stage III melanoma patients.

Such data are critical to determine which patients would benefit from adjuvant treatment. Current regimens provide modest benefits coupled with very common and significant toxicities that result in decreased quality of life. High dose interferon alpha raises recurrence free survival (RFS) by approximately 11% in 5 years but has associated severe toxicities in the large majority of patients including granulocytopenias, liver toxicity, thyroid toxicity, and severe fatigue^13^. Ipilimumab, which increases 5 year RFS, distant metastasis free survival, and OS by approximately 10%, has nearly all patients suffer some adverse events (AEs) with half suffering significant grade 3 or 4 AEs of dermatologic, gastrointestinal, endocrine, and hepatic failure^14^. Because of the frequency and severity of these toxicities, NLR as a prognostic factor would allow for better selection of patients for adjuvant treatment.

Our study suggests that females have better outcomes and survival than male patients with stage III melanoma. This finding is consistent with several prior studies including one that found there were more females in NLR < 5 group compared with NLR ≥ 5 group (35.6% vs 29.5%), which evaluated the predictive value of NLR for survival in patients with colorectal liver cancer^8^. Even though one has yet to discover the main reason for improved melanoma survival among females compared to males, many studies have suggested potential possibilities including their better self-detection rates, closer follow up to health-care providers, and are more likely to engage in preventative behaviors^15–17^. Our study suggests that the survival advantage is in part due to having a low NLR. In addition, the WBC count in the low NLR group is lower compared to that in the high NLR group. This finding might be explained by patients with high NLR have more inflammatory response.

Because NLR integrates information on the inflammatory milieu and physiological stress, many studies indicated it is superior to other leukocyte parameters such as leukocyte counts for predicting prognosis of different disease^18–21^. In addition, NLR is more stable when it is used to predict the prognosis because other leukocyte counts could be impacted by many factors such as smoking, weight, hemoglobin, and diastolic blood pressure.

In this study, we used the NLR with most significant log-rank test p-value as the optimal cutoff. The optimal cutoff point used in this study was 2.5, which is different from the values used in prior studies. Lino-Silva used 2 as the NLR cutoff to predict overall survival in melanoma patients. They did not differentiate between different stages when they determined the cutoff using operating characteristic (ROC) curve analysis^4^. Bhat *et al*. also used ROC curve analysis to determine the optimal cutoff of 5 in patients with Stage III or IV unresectable melanoma^22^. Zaragoza J *et al*. used 4, also determined using ROC analysis, as the cutoff of NLR to evaluate the usefulness of NLR for predicting overall survival in stage III or IV melanoma patients^10^. The difference of cutoff point could be because the study population was dissimilar between the studies. The study population in our study is stage III melanoma patients without histologically proven recurrence. In contrast, prior studies did not differentiate stage III from other stages. This difference may be also due to the methods used to determine the optimal cutoff. Since ROC curves do not account for time to event and right censoring, the method used in our study is more appropriate for prognostic questions^23^.

There are several limitations of this study. First, this is a single-center retrospective cohort study. The majority of the patients were from Utah where there is approximately 66% of sun exposure annually and prolonged sun exposure has been suggested to be an important risk factor for developing melanoma^11^. The results from this study need to be applied to other population with cautiousness. Second, we only evaluated the DFS due to the limited follow-up data. Evaluating the long-term OS might give us a deeper insight into the usefulness of NLR for predicting prognosis and recurrence in patients with melanoma. Third, this study only focused on stage III melanoma patients. The predictive value of NLR might be different in patients diagnosed with other stages of melanoma.

In conclusion, the NLR is a strong predictor of disease recurrence in patients with stage III melanoma. The conclusion should be validated by larger prospective studies. Especially, the association between long-term OS and the NLR needs to be further studied.

## Methods

This retrospective study utilized a cohort of patients diagnosed with stage III melanoma treated at Huntsman Cancer Institute, University of Utah, from May 2002 to March 2016. Inclusion criteria included patients with stage III melanoma, without histologically proven recurrence, an absolute neutrophil count (ANC) and an absolute lymphocyte count (ALC) available at baseline. A total of 107 patients who met the inclusion criteria were included in this study. The optimal cutoff of NLR was determined by the significance of log-rank tests^24^. A total of 97 log-rank tests were conducted to find the optimal cutoff. DFS was assessed using the Kaplan–Meier method, and univariable and multivariable Cox models were applied to evaluate the association between predictive value of NLR and DFS.

Data were obtained from clinical medical records, which include demographic variables, site of melanoma, date of diagnosis, adjuvant therapy, radiation therapy, the tumor size (T stage), lymph node status (N stage) and mitotic count. The medical records for the patients were reviewed by two investigators independently. The study was approved by the Institutional Review Board (IRB) at University of Utah, and a waiver of consent was permitted. All analyses were conducted in compliance with the approved study protocol. The datasets generated and analysed during the study are available from the corresponding authors on reasonable request.

To determine the optimal cutoff point of NLR in our study, we performed 97 log-rank tests based on all possible NLR values of this cohort. The optimal cutoff was defined as the NLR value with the smallest p-value of log-rank tests. After determining the optimal NLR cutoff, we classified patients into two groups based on their NLR values. Patients were categorized as having high NLR if their NLR values were above or equal to the cutoff point, and individuals were categorized as having low NLR if their NLR values were below the optimal cutoff value. Distributions of continuous variables were summarized as median and interquartile range (IQR), and distributions of categorical variables were described as frequencies and percentages. Rank sum test and chi-squared test were used to compare continuous variables and categorical variables, respectively. DFS was assessed using the KM method. In addition, univariable and multivariable Cox proportional hazard models were used to evaluate the association between NLR cutoff value and DFS. The multivariable model controlled for age of diagnosis, gender, site of melanoma, stage IIIA, B or C status, lymph node status (N stage), adjuvant therapy, the tumor size (T stage), ulceration status, white blood cell counts, tumor-infiltrating lymphocytes (TILs), and the Breslow depth. HRs and their corresponding 95% CIs were estimated. The proportional-hazards assumption was checked by scaled Schoenfeld residuals. The statistical significance for P value was designated as less than 0.05. All the analyses were conducted using STATA 14.0 (Stata, College Station, TX) and R software version 3.4.0.
